# Supplementary material for: Investigating the role of climate-related disasters in the relationship between food insecurity and mental health for youth aged 15–24 in 142 countries
Source: PLOS Glob Public Health. 2022 Sep 7;2(9):e0000560. doi: 10.1371/journal.pgph.0000560 (PMC10021754; doi:10.1371/journal.pgph.0000560)
Supplement: S1 Text — (DOCX) [file pgph.0000560.s001.docx]

Food Insecurity Experiences Scale (FIES) individual questions and corresponding food insecurity constructs.

| **Question (in the last 12 months, was there a time when…)** | **Food Insecurity Construct** |
| --- | --- |
| (1) you were worried you would not have enough food to eat because of a lack of money or other resources? | Uncertainty and worry about food (mild) |
| (2) you were unable to eat healthy and nutritious food because of a lack of money or other resources? | Inadequate food quality (mild) |
| (3) you ate only a few kinds of foods because of a lack of money or other resources? | Inadequate food quality (mild) |
| (4) you had to skip a meal because there was not enough money or other resources? | Insufficient food quantity (moderate) |
| (5) you ate less than you thought you should because of a lack of money or other resources? | Insufficient food quantity (moderate) |
| (6) your household ran out of food because of a lack of money or other resources? | Insufficient food quantity (moderate) |
| (7) you were hungry but did not eat because there was not enough money or other resources for food? | Insufficient food quantity (severe) |
| (8) you went without eating for a whole day because of a lack of money or other resources? | Insufficient food quantity (severe) |

Daily Experience Index (DEI) individual questions and corresponding wellbeing constructs.

| **Question** | **Wellbeing Construct** |
| --- | --- |
| (1) Did you feel well-rested yesterday? | Positive wellbeing |
| (2) Were you treated with respect all day yesterday? | Positive wellbeing |
| (3) Did you smile or laugh a lot yesterday? | Positive wellbeing |
| (4) Did you learn or do something interesting yesterday? | Positive wellbeing |
| (5) Did you experience the following feelings during a lot of the day yesterday? How about enjoyment? | Positive wellbeing |
| (6) Did you experience the following feelings during a lot of the day yesterday? How about physical pain? | Negative wellbeing |
| (7) Did you experience the following feelings during a lot of the day yesterday? How about worry? | Negative wellbeing |
| (8) Did you experience the following feelings during a lot of the day yesterday? How about sadness? | Negative wellbeing |
| (9) Did you experience the following feelings during a lot of the day yesterday? How about stress? | Negative wellbeing |
| (10) Did you experience the following feelings during a lot of the day yesterday? How about anger? | Negative wellbeing |

Histogram showing the distribution of the potential effect modifier variable, total number of climate-related disaster deaths per 1,000,000 population from 2015-2017.


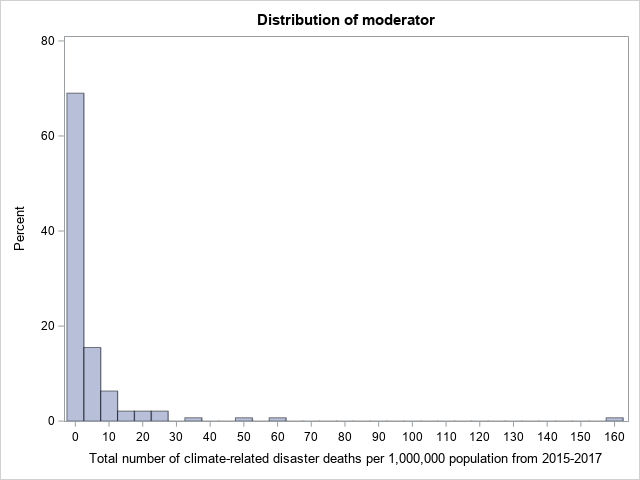


List of all countries (n=142) included in the present analysis of the 2017 cycle of the Gallup World Poll, with their corresponding mode of data collection, sample size, youth sample size, country income level and United Nations (UN) sub-region. Adapted from the Gallup World Poll Dataset Details.

| **Country*** | **Mode of Data Collection** | **Total Sample Size** | **Youth Sample Size**** | **Country Income Level***** | **UN**  **Sub-Region****** |
| --- | --- | --- | --- | --- | --- |
| Afghanistan | Face-to-face | 1,000 | 282 | Low | Southern Asia |
| Albania | Face-to-face | 1,000 | 177 | Middle | Southern Europe |
| Algeria | Face-to-face | 1,016^b^ | 231 | Middle | Northern Africa |
| Argentina | Face-to-face | 1,000^a^ | 173 | Middle | Latin America and the Caribbean |
| Armenia | Face-to-face | 1,000 | 156 | Middle | Western Asia |
| Australia | Landline and mobile telephone | 1,003 | 47 | High | Australia and New Zealand |
| Austria | Landline and mobile telephone | 1,000 | 124 | High | Western Europe |
| Azerbaijan | Face-to-face | 1,000^b^ | 197 | Middle | Western Asia |
| Bahrain | Landline and mobile telephone | 1,064 | 162 | High | Western Asia |
| Bangladesh | Face-to-face | 1,000^a^ | 228 | Middle | Southern Asia |
| Belarus | Face-to-face | 1,053 | 139 | Middle | Eastern Europe |
| Belgium | Landline and mobile telephone | 1,001 | 89 | High | Western Europe |
| Benin | Face-to-face | 1,000 | 350 | Middle | Sub-Saharan Africa |
| Bolivia | Face-to-face | 1,000 | 283 | Middle | Latin America and the Caribbean |
| Bosnia Herzegovina | Face-to-face | 1,000 | 128 | Middle | Southern Europe |
| Botswana | Face-to-face | 1,000 | 225 | Middle | Sub-Saharan Africa |
| Bulgaria | Face-to-face | 1,000 | 53 | Middle | Eastern Europe |
| Burkina Faso | Face-to-face | 1,000 | 329 | Low | Sub-Saharan Africa |
| Cambodia | Face-to-face | 1,600^a^ | 278 | Middle | South-eastern Asia |
| Cameroon | Face-to-face | 1,000^b^ | 313 | Middle | Sub-Saharan Africa |
| Canada | Landline and mobile telephone | 1,005 | 85 | High | Northern America |
| Central African Republic | Face-to-face | 1,000^c^ | 311 | Low | Sub-Saharan Africa |
| Chad | Face-to-face | 1,000^b^ | 298 | Low | Sub-Saharan Africa |
| Chile | Face-to-face | 1,040^a^ | 174 | High | Latin America and the Caribbean |
| China | Landline and mobile telephone and Face-to-face | 4,141^a^ | 322 | Middle | Eastern Asia |
| Colombia | Face-to-face | 1,000 | 224 | Middle | Latin America and the Caribbean |
| Congo Brazzaville | Face-to-face | 1,000 | 264 | Middle | Sub-Saharan Africa |
| Congo Kinshasa | Face-to-face | 1,000^c^ | 330 | Low | Sub-Saharan Africa |
| Costa Rica | Face-to-face | 1,000 | 171 | Middle | Latin America and the Caribbean |
| Croatia | Face-to-face | 1,000 | 219 | High | Southern Europe |
| Cyprus | Landline and mobile telephone | 1,008 | 156 | High | Western Asia |
| Czech Republic | Face-to-face | 1,000 | 97 | High | Eastern Europe |
| Denmark | Landline and mobile telephone | 1,000 | 122 | High | Northern Europe |
| Dominican Republic | Face-to-face | 1,000 | 199 | Middle | Latin America and the Caribbean |
| Ecuador | Face-to-face | 1,000 | 222 | Middle | Latin America and the Caribbean |
| Egypt | Face-to-face | 1,000^a^ | 178 | Middle | Northern Africa |
| El Salvador | Face-to-face | 1,000 | 198 | Middle | Latin America and the Caribbean |
| Estonia | Face-to-face | 1,000 | 106 | High | Northern Europe |
| Ethiopia | Face-to-face | 1,000 | 289 | Low | Sub-Saharan Africa |
| Finland | Mobile telephone | 1,000 | 45 | High | Northern Europe |
| France | Landline and mobile telephone | 1,000 | 66 | High | Western Europe |
| Gabon | Face-to-face | 1,000 | 246 | Middle | Sub-Saharan Africa |
| Gambia | Face-to-face | 1,000 | 314 | Low | Sub-Saharan Africa |
| Georgia | Face-to-face | 1,000 | 97 | Middle | Western Asia |
| Germany | Landline and mobile telephone | 1,000 | 137 | High | Western Europe |
| Ghana | Face-to-face | 1,000 | 313 | Middle | Sub-Saharan Africa |
| Greece | Face-to-face | 1,000 | 103 | High | Southern Europe |
| Guatemala | Face-to-face | 1,000 | 296 | Middle | Latin America and the Caribbean |
| Guinea | Face-to-face | 1,000 | 330 | Low | Sub-Saharan Africa |
| Haiti | Face-to-face | 504 | 130 | Low | Latin America and the Caribbean |
| Honduras | Face-to-face | 1,000 | 273 | Middle | Latin America and the Caribbean |
| Hong Kong | Landline and mobile telephone | 1,007 | 146 | High | Eastern Asia |
| Hungary | Face-to-face | 1,000 | 69 | High | Eastern Europe |
| Iceland | Landline and mobile telephone | 500 | 53 | High | Northern Europe |
| India | Face-to-face | 3,000^a^ | 679 | Middle | Southern Asia |
| Indonesia | Face-to-face | 1,000 | 185 | Middle | South-eastern Asia |
| Iran | Landline and mobile telephone | 1,000 | 147 | Middle | Southern Asia |
| Iraq | Landline and mobile telephone | 1,000 | 201 | Middle | Western Asia |
| Ireland | Landline and mobile telephone | 1,000 | 115 | High | Northern Europe |
| Israel | Face-to-face | 1,000 | 155 | High | Western Asia |
| Italy | Landline and mobile telephone | 1,000 | 102 | High | Southern Europe |
| Ivory Coast | Face-to-face | 1,000 | 274 | Middle | Sub-Saharan Africa |
| Jamaica | Face-to-face | 504 | 87 | Middle | Latin America and the Caribbean |
| Japan | Landline and mobile telephone | 1,002^a^ | 46 | High | Eastern Asia |
| Jordan | Face-to-face | 1,012 | 273 | Middle | Western Asia |
| Kazakhstan | Face-to-face | 1,000 | 132 | Middle | Central Asia |
| Kenya | Face-to-face | 1,000^a^ | 356 | Middle | Sub-Saharan Africa |
| Kosovo | Face-to-face | 1,000 | 221 | Middle | Southern Europe |
| Kuwait | Landline and mobile telephone | 1,000 | 84 | High | Western Asia |
| Kyrgyzstan | Face-to-face | 1,000 | 160 | Middle | Central Asia |
| Laos | Face-to-face | 1,000^b^ | 219 | Middle | South-eastern Asia |
| Latvia | Face-to-face | 1,002 | 70 | High | Northern Europe |
| Lebanon | Face-to-face | 1,000^b^ | 137 | Middle | Western Asia |
| Lesotho | Face-to-face | 1,000 | 253 | Middle | Sub-Saharan Africa |
| Liberia | Face-to-face | 1,000 | 367 | Low | Sub-Saharan Africa |
| Libya | Mobile telephone | 1,007 | 215 | Middle | Northern Africa |
| Lithuania | Face-to-face | 1,000 | 199 | High | Northern Europe |
| Luxembourg | Landline and mobile telephone | 1,000 | 156 | High | Western Europe |
| Madagascar | Face-to-face | 1,000^c^ | 253 | Low | Sub-Saharan Africa |
| Malawi | Face-to-face | 1,000 | 349 | Low | Sub-Saharan Africa |
| Mali | Face-to-face | 1,000^c^ | 302 | Low | Sub-Saharan Africa |
| Malta | Landline and mobile telephone | 1,004 | 96 | High | Southern Europe |
| Mauritius | Landline and mobile telephone | 1,000 | 165 | High | Sub-Saharan Africa |
| Mexico | Face-to-face | 1,000 | 186 | Middle | Latin America and the Caribbean |
| Mongolia | Face-to-face | 1,000 | 181 | Middle | Eastern Asia |
| Montenegro | Face-to-face | 1,000^a^ | 161 | Middle | Southern Europe |
| Morocco | Face-to-face | 1,006^a^ | 204 | Middle | Northern Africa |
| Mozambique | Face-to-face | 1,000 | 340 | Low | Sub-Saharan Africa |
| Myanmar | Face-to-face | 1,600^a^ | 267 | Middle | South-eastern Asia |
| Namibia | Face-to-face | 1,000 | 361 | Middle | Sub-Saharan Africa |
| Nepal | Face-to-face | 1,000 | 205 | Middle | Southern Asia |
| Netherlands | Landline and mobile telephone | 1,001 | 85 | High | Western Europe |
| New Zealand | Landline and mobile telephone | 1,001 | 82 | High | Australia and New Zealand |
| Nicaragua | Face-to-face | 1,000 | 230 | Middle | Latin America and the Caribbean |
| Niger | Face-to-face | 1,000 | 330 | Low | Sub-Saharan Africa |
| Nigeria | Face-to-face | 1,000^a^ | 322 | Middle | Sub-Saharan Africa |
| North Macedonia | Face-to-face | 1,008 | 96 | Middle | Southern Europe |
| Norway | Landline and mobile telephone | 1,000 | 78 | High | Northern Europe |
| Pakistan | Face-to-face | 1,600^a^ | 408 | Middle | Southern Asia |
| Palestine | Face-to-face | 1,000^a^ | 272 | Middle | Western Asia |
| Panama | Face-to-face | 1,000 | 220 | High | Latin America and the Caribbean |
| Paraguay | Face-to-face | 1,000 | 198 | Middle | Latin America and the Caribbean |
| Peru | Face-to-face | 1,000 | 248 | Middle | Latin America and the Caribbean |
| Philippines | Face-to-face | 1,000 | 198 | Middle | South-eastern Asia |
| Poland | Face-to-face | 1,000 | 116 | High | Eastern Europe |
| Portugal | Landline and mobile telephone | 1,000 | 63 | High | Southern Europe |
| Romania | Face-to-face | 1,001 | 76 | High | Eastern Europe |
| Russia | Face-to-face | 2,000^b^ | 242 | Middle | Eastern Europe |
| Rwanda | Face-to-face | 1,000 | 239 | Low | Sub-Saharan Africa |
| Saudi Arabia | Landline and mobile telephone | 1,002 | 242 | High | Western Asia |
| Senegal | Face-to-face | 1,000 | 319 | Middle | Sub-Saharan Africa |
| Serbia | Face-to-face | 1,000 | 83 | Middle | Southern Europe |
| Sierra Leone | Face-to-face | 1,000 | 335 | Low | Sub-Saharan Africa |
| Singapore | Face-to-face | 1,000^b^ | 109 | High | South-eastern Asia |
| Slovakia | Face-to-face | 1,000 | 92 | High | Eastern Europe |
| Slovenia | Landline and mobile telephone | 1,000 | 45 | High | Southern Europe |
| South Africa | Face-to-face | 1,000 | 259 | Middle | Sub-Saharan Africa |
| South Korea | Landline and mobile telephone | 1,000 | 91 | High | Eastern Asia |
| South Sudan | Face-to-face | 1,000^c^ | 369 | Low | Sub-Saharan Africa |
| Spain | Landline and mobile telephone | 1,000 | 120 | High | Southern Europe |
| Sri Lanka | Face-to-face | 1,104 | 166 | Middle | Southern Asia |
| Sweden | Landline and mobile telephone | 1,000 | 101 | High | Northern Europe |
| Switzerland | Landline and mobile telephone | 1,000 | 118 | High | Western Europe |
| Taiwan | Landline and mobile telephone | 1,000 | 112 | High | Eastern Asia |
| Tajikistan | Face-to-face | 1,000 | 248 | Low | Central Asia |
| Tanzania | Face-to-face | 1,000 | 276 | Middle | Sub-Saharan Africa |
| Thailand | Face-to-face | 1,000^a^ | 91 | Middle | South-eastern Asia |
| Togo | Face-to-face | 1,000 | 349 | Low | Sub-Saharan Africa |
| Trinidad and Tobago | Face-to-face | 504 | 47 | High | Latin America and the Caribbean |
| Tunisia | Face-to-face | 1,001 | 182 | Middle | Northern Africa |
| Turkey | Face-to-face | 1,000 | 179 | Middle | Western Asia |
| Uganda | Face-to-face | 1,000 | 357 | Low | Sub-Saharan Africa |
| Ukraine | Face-to-face | 1,000^b^ | 75 | Middle | Eastern Europe |
| United Arab Emirates | Landline and mobile telephone | 1,850 | 319 | High | Western Asia |
| United Kingdom | Landline and mobile telephone | 1,000 | 124 | High | Northern Europe |
| United States | Landline and mobile telephone | 1,013 | 87 | High | Northern America |
| Uruguay | Face-to-face | 1,000 | 146 | High | Latin America and the Caribbean |
| Uzbekistan | Face-to-face | 1,000 | 185 | Middle | Central Asia |
| Venezuela | Face-to-face | 1,000^a^ | 166 | Middle | Latin America and the Caribbean |
| Yemen | Face-to-face | 1,000^c^ | 288 | Low | Western Asia |
| Zambia | Face-to-face | 1,000 | 376 | Middle | Sub-Saharan Africa |
| Zimbabwe | Face-to-face | 1,000 | 283 | Middle | Sub-Saharan Africa |
| **TOTAL** |  | **149,164** | **28,292** |  |  |

*Note that while the Poll collected data from n=148 countries, the following six were excluded due to large amounts of missing data for variables of interest: Brazil (n=1,000, n=167 youth), Moldova (n=1,000, n=131 youth), Mauritania (n=1,000, n=299 youth), Maldives (n=1,000, n=200 youth), Vietnam (n=1,002, n=133 youth), Turkmenistan (n=1,000, n=263 youth).

**Youth sample ages 15-24 years. Note that n=194 youth were excluded from this analysis due to missing outcome data.

***The World Bank Atlas method was used to determine country income level (the 2019 gross national income per capita was ≤ $1,035 for low-income countries, $1,036-$12,535 for middle-income countries, and ≥$12,536 for high-income countries).

****United Nations (UN) sub-regions: <https://unstats.un.org/unsd/methodology/m49/overview/>.

^a^Percentage of total population excluded due to physical inaccessibility or security concerns <10%.

^b^Percentage of total population excluded due to physical inaccessibility or security concerns 10-20%.

^c^Percentage of total population excluded due to physical inaccessibility or security concerns >20%.

Histogram showing the distribution of poor mental health scores for the 2017 Gallup World Poll study sample (n=28,292 youth in n=142 countries). Based on weighted data.

**
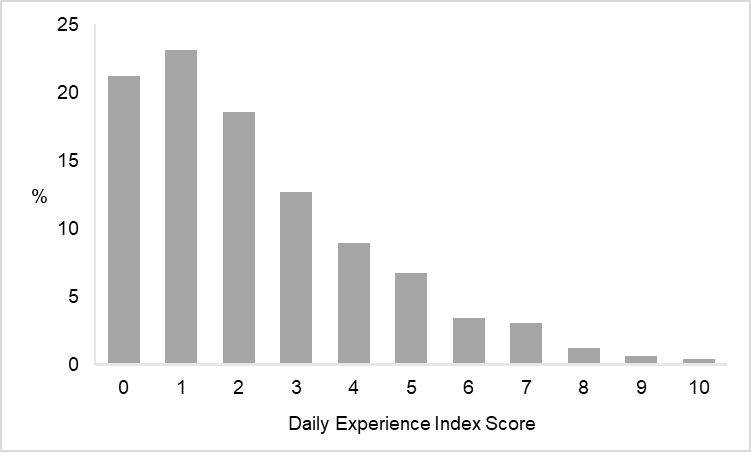
**

Characteristics of the included climate-related disasters from the International Disaster Database (n=828) that occurred between 2015-2017 in the 142 countries from the Gallup World Poll sample.

| **Characteristic** |  |
| --- | --- |
|  | *n (%)* |
| Disasters by country income level^a^  Low-income countries  Middle-income countries  High-income countries | 94 (11.4%)  503 (60.8%)  231 (27.9%) |
| Disasters by country (top 5)  China  United States  India  Philippines  Indonesia  Other countries | 87 (10.5%)  79 (9.5%)  52 (6.3%)  37 (4.5%)  33 (4.0%)  540 (65.2%) |
| Disasters by year  2015  2016  2017 | 301 (36.4%)  257 (31.0%)  270 (32.6%) |
| Disaster subgroup  Meteorological  Hydrological  Climatological | 307 (37.1%)  450 (54.4%)  71 (8.6%) |
| Disaster type  Drought  Extreme temperature  Flood  Landslide  Storm  Wildfire | 34 (4.1%)  32 (3.9%)  395 (47.7%)  55 (6.6%)  275 (33.2%)  37 (4.5%) |

^a^The World Bank Atlas method was used to determine country income level (the 2019 gross national income per capita was ≤ $1,035 for low-income countries, $1,036-$12,535 for middle-income countries, and ≥$12,536 for high-income countries).

Multivariate multilevel negative binomial regression models examining the association between food insecurity (Food Insecurity Experiences Scale) and poor mental health (Daily Experience Index score) among n=28,292 youth from the 2017 Gallup World Poll survey, by country income level.

|  | **LICs** | **MICs** | **HICs** |
| --- | --- | --- | --- |
| **Factor** | *RR (95% CI)* | *RR (95% CI)* | *RR (95% CI)* |
| Food insecurity  None or mild  Moderate  Severe | 1 (ref)  1.31 (1.25-1.38)***  1.53 (1.40-1.66)*** | 1 (ref)  1.37 (1.31-1.43)***  1.61 (1.54-1.68)*** | 1 (ref)  1.45 (1.30-1.62)***  1.74 (1.57-1.94)*** |
| Gender  Male  Female | 1 (ref)  0.98 (0.93-1.03) | 1 (ref)  1.04 (1.00-1.08)* | 1 (ref)  1.03 (0.98-1.10) |
| Urbanicity  Urban  Suburban  Small village  Rural | 1 (ref)  0.98 (0.93-1.03)  0.99 (0.94-1.04)  1.04 (0.96-1.13) | 1 (ref)  0.96 (0.91-1.02)  0.98 (0.94-1.01)  0.99 (0.95-1.03) | 1 (ref)  1.05 (0.96-1.14)  0.95 (0.89-1.02)  1.06 (0.92-1.23) |
| Marital status  Single  Married  Separated  Divorced  Widowed  Domestic partner | 1 (ref)  1.05 (1.00-1.11)*  1.19 (1.04-1.36)*  1.10 (0.92-1.31)  1.6 (1.32-1.94)***  0.97 (0.86-1.10) | 1 (ref)  1.06 (1.00-1.14)  1.12 (0.94-1.35)  1.08 (0.83-1.39)  1.37 (1.06-1.77)*  1.07 (1.01-1.13)* | 1 (ref)  1.09 (0.97-1.22)  1.16 (0.85-1.58)  1.30 (1.14-1.49)**  0.96 (0.81-1.14)  0.97 (0.90-1.06) |
| Education  Tertiary  Secondary  Elementary or less | 1 (ref)  1.03 (0.92-1.15)  1.08 (0.96-1.22) | 1 (ref)  1.01 (0.94-1.08)  1.03 (0.95-1.12) | 1 (ref)  1.10 (1.02-1.19)*  1.06 (0.93-1.21) |
| Employment  Full-time (employer)  Full-time (self-employed)  Part-time (seeking full-time)  Part-time (not seeking full-time)  Unemployed  Out of workforce | 1 (ref)  0.98 (0.93-1.04)  0.99 (0.91-1.07)  0.99 (0.91-1.08)  1.12 (1.03-1.22)**  0.96 (0.88-1.05) | 1 (ref)  0.96 (0.90-1.03)  0.91 (0.86-0.96)**  0.91 (0.86-0.97)**  0.95 (0.90-1.01)  0.83 (0.78-0.88)*** | 1 (ref)  1.13 (0.96-1.32)  0.99 (0.89-1.11)  1.01 (0.93-1.10)  1.09 (0.99-1.21)  0.90 (0.84-0.97)* |
| Number of children in household <15 years | 1.00 (1.00-1.01) | 1.01 (1.00-1.02)** | 1.01 (0.99-1.03) |
| Age | 1.02 (1.01-1.02)** | 1.02 (1.01-1.03)*** | 1.03 (1.02-1.04)*** |
| Log annual household income | 0.97 (0.95-0.98)** | 0.96 (0.94-0.98)** | 0.95 (0.91-0.99)* |
| Disaster severity^a^  Low  High | 1 (ref)  0.97 (0.84-1.13) | 1 (ref)  0.99 (0.81-1.22) | 1 (ref)  1.16 (0.89-1.52) |

**Notes:** The World Bank Atlas method was used to determine country income level (the 2019 gross national income per capita was ≤ $1,035 for low-income countries, $1,036-$12,535 for middle-income countries, and ≥$12,536 for high-income countries). Models present relative risk (RR) estimates with 95% confidence intervals (CI). All models were adjusted for clustering at the country level (random effect).

^a^Disaster severity was measured at the country level. High disaster severity was defined as those countries in the top 10% of total number of climate-related disaster deaths per 1,000,000 population between the years of 2015-2017. Low disaster severity was defined as those countries in the bottom 90% of total number of climate-related disaster deaths per 1,000,000 population between the years of 2015-2017.

**Abbreviations:** high-income countries (HICs), low-income countries (LICs), middle-income countries (MICs)

*p<0.05, **p<0.01, ***p<0.0001

Multivariate multilevel negative binomial regression model examining the association between food insecurity (Food Insecurity Experiences Scale) and poor mental health (Daily Experience Index score) among n=28,292 youth from the 2017 Gallup World Poll survey. Note that the interaction between food insecurity and disaster severity was not statistically significant (p=0.5362).

|  | **Multivariate Model** | |
| --- | --- | --- |
| **Factor** | *RR (95% CI)* | *p* |
| Food insecurity  None or mild  Moderate  Severe | 1 (ref)  1.35 (1.29-1.42)  1.62 (1.53-1.71) | <0.0001  <0.0001 |
| Gender  Male  Female | 1 (ref)  1.03 (1.00-1.05) | 0.0689 |
| Urbanicity  Urban  Suburban  Small village  Rural | 1 (ref)  0.99 (0.95-1.03)  0.97 (0.95-1.00)  1.01 (0.97-1.05) | 0.6911  0.0534  0.7033 |
| Marital status  Single  Domestic partner  Married  Separated  Divorced  Widowed | 1 (ref)  1.03 (0.98-1.08)  1.06 (1.01-1.10)  1.15 (1.03-1.28)  1.13 (0.99-1.28)  1.34 (1.13-1.58) | 0.2767  0.0093  0.0149  0.0641  0.0005 |
| Education  Tertiary  Secondary  Elementary or less | 1 (ref)  1.04 (0.99-1.09)  1.06 (1.00-1.13) | 0.1681  0.0400 |
| Employment  Full-time (employer)  Full-time (self-employed)  Part-time (seeking full-time)  Part-time (not seeking full-time)  Unemployed  Out of workforce | 1 (ref)  0.97 (0.93-1.02)  0.94 (0.90-0.98)  0.95 (0.91-0.99)  1.01 (0.97-1.06)  0.87 (0.83-0.91) | 0.2782  0.0059  0.0198  0.5549  <0.0001 |
| Number of children in household <15 years | 1.01 (1.00-1.01) | 0.0034 |
| Age | 1.02 (1.02-1.03) | <0.0001 |
| Log annual household income | 0.96 (0.95-0.97) | <0.0001 |
| Disaster severity^a^  Low  High | 1 (ref)  1.05 (0.93-1.18) | 0.4483 |

**Notes:** Model presents relative risk (RR) estimates with 95% confidence intervals (CI). Model was adjusted for clustering at the country level (random effect).

^a^Disaster severity was measured at the country level. High disaster severity was defined as those countries in the top 10% of total number of climate-related disaster deaths per 1,000,000 population between the years of 2015-2017. Low disaster severity was defined as those countries in the bottom 90% of total number of climate-related disaster deaths per 1,000,000 population between the years of 2015-2017.

Multivariate multilevel negative binomial regression model examining the association between food insecurity (Food Insecurity Experiences Scale) and poor mental health (Daily Experience Index score) among n=28,292 youth from the 2017 Gallup World Poll survey. Note that the interaction between food insecurity and disaster severity was not statistically significant (p=0.1098).

|  | **Multivariate Model** | |
| --- | --- | --- |
| **Factor** | *RR (95% CI)* | *p* |
| Food insecurity  None or mild  Moderate  Severe | 1 (ref)  1.36 (1.32-1.41)  1.59 (1.53-1.66) | <0.0001  <0.0001 |
| Gender  Male  Female | 1 (ref)  1.03 (1.00-1.06) | 0.0363 |
| Urbanicity  Urban  Suburban  Small village  Rural | 1 (ref)  0.99 (0.95-1.03)  0.97 (0.95-1.00)  1.00 (0.97-1.04) | 0.6004  0.0344  0.8839 |
| Marital status  Single  Married  Separated  Divorced  Widowed  Domestic partner | 1 (ref)  1.05 (1.01-1.10)  1.17 (1.05-1.31)  1.12 (0.98-1.27)  1.35 (1.15-1.60)  1.03 (0.98-1.08) | 0.0160  0.0062  0.0895  0.0004  0.2543 |
| Education  Tertiary  Secondary  Elementary or less | 1 (ref)  1.05 (1.00-1.10)  1.08 (1.02-1.14) | 0.0701  0.0118 |
| Employment  Full-time (employer)  Full-time (self-employed)  Part-time (seeking full-time)  Part-time (not seeking full-time)  Unemployed  Out of workforce | 1 (ref)  0.97 (0.92-1.01)  0.93 (0.89-0.97)  0.94 (0.90-0.98)  1.00 (0.96-1.05)  0.86 (0.83-0.90) | 0.1463  0.0019  0.0071  0.8407  <0.0001 |
| Number of children in household <15 years | 1.01 (1.00-1.01) | 0.0028 |
| Age | 1.02 (1.02-1.03) | <0.0001 |
| Log annual household income | 0.96 (0.95-0.97) | <0.0001 |
| Disaster severity^a^ | 1.00 (1.00-1.00) | 0.0015 |

**Notes:** Model presents relative risk (RR) estimates with 95% confidence intervals (CI). Model was adjusted for clustering at the country level (random effect).

^a^Disaster severity was measured at the country level. It was represented as a continuous frequency variable: total number of climate-related disasters per 1,000,000 km^2^ country surface area between the years of 2015-2017.

Multivariate multilevel negative binomial regression model examining the association between food insecurity (Food Insecurity Experiences Scale) and poor mental health (Daily Experience Index score) among n=28,292 youth from the 2017 Gallup World Poll survey. Note that the interaction between food insecurity and disaster severity was not statistically significant (p=0.1923).

|  | **Multivariate Model** | |
| --- | --- | --- |
| **Factor** | *RR (95% CI)* | *p* |
| Food insecurity  None or mild  Moderate  Severe | 1 (ref)  1.40 (1.34-1.47)  1.66 (1.57-1.75) | <0.0001  <0.0001 |
| Gender  Male  Female | 1 (ref)  1.03 (1.00-1.05) | 0.0695 |
| Urbanicity  Urban  Suburban  Small village  Rural | 1 (ref)  0.99 (0.95-1.03)  0.97 (0.95-1.00)  1.01 (0.97-1.05) | 0.6723  0.0515  0.7043 |
| Marital status  Single  Married  Separated  Divorced  Widowed  Domestic partner | 1 (ref)  1.06 (1.01-1.10)  1.14 (1.02-1.28)  1.13 (0.99-1.29)  1.34 (1.13-1.58)  1.03 (0.98-1.08) | 0.0096  0.0191  0.0614  0.0006  0.2818 |
| Education  Tertiary  Secondary  Elementary or less | 1 (ref)  1.04 (0.98-1.09)  1.06 (1.00-1.13) | 0.1787  0.0438 |
| Employment  Full-time (employer)  Full-time (self-employed)  Part-time (seeking full-time)  Part-time (not seeking full-time)  Unemployed  Out of workforce | 1 (ref)  0.97 (0.93-1.02)  0.94 (0.90-0.98)  0.95 (0.91-0.99)  1.01 (0.97-1.06)  0.87 (0.83-0.91) | 0.2840  0.0060  0.0203  0.5534  <0.0001 |
| Number of children in household <15 years | 1.01 (1.00-1.01) | 0.0033 |
| Age | 1.02 (1.02-1.03) | <0.0001 |
| Log annual household income | 0.96 (0.95-0.97) | <0.0001 |
| Disaster severity^a^  Low  High | 1 (ref)  0.96 (0.86-1.07) | 0.4506 |

**Notes:** Model presents relative risk (RR) estimates with 95% confidence intervals (CI). Model was adjusted for clustering at the country level (random effect).

^a^Disaster severity was measured at the country level. High disaster severity was defined as those countries in the top 10% of total number of climate-related disaster deaths per 1,000,000 population in 2017. Low disaster severity was defined as those countries in the bottom 90% of total number of climate-related disaster deaths per 1,000,000 population in 2017.
