# Supplementary material for: Investigating the role of climate-related disasters in the relationship between food insecurity and mental health for youth aged 15–24 in 142 countries
Source: PLOS Glob Public Health. 2022 Sep 7;2(9):e0000560. doi: 10.1371/journal.pgph.0000560 (PMC10021754; doi:10.1371/journal.pgph.0000560)
Supplement: Attachment — Submitted filename: Response to Reviewers.docx [file pgph.0000560.s002.docx]

| **Reviewer Comment** | **Response** |
| --- | --- |
| *Journal Requirements* | |
| 1. Please provide us with a direct link to the base layer of the map used in Figure 2 and ensure this location is also included in the figure legend.  Please note that, because all PLOS articles are published under a CC BY license (creativecommons.org/licenses/by/4.0/), we cannot publish proprietary maps such as Google Maps, Mapquest or other copyrighted maps. If your map was obtained from a copyrighted source please amend the figure so that the base map used is from an openly available source.  Please note that only the following CC BY licences are compatible with PLOS licence: CC BY 4.0, CC BY 2.0  and CC BY 3.0, meanwhile such licences as CC BY-ND 3.0 and others are not compatible due to additional restrictions. If you are unsure whether you can use a map or not, please do reach out and we will be able to help you.  The following websites are good examples of where you can source open access or public domain maps:  * U.S. Geological Survey (USGS) - All maps are in the public domain. (http://www.usgs.gov)  * PlaniGlobe - All maps are published under a Creative Commons license so please cite “PlaniGlobe, http://www.planiglobe.com, CC BY 2.0” in the image credit after the caption. (http://www.planiglobe.com/?lang=enl)  * Natural Earth - All maps are public domain. (http://www.naturalearthdata.com/about/terms-of-use/) | We have included a direct link to the base layer of the map in the figure legend (lines 310-311). Based on the requirements of Esri (<https://resources.esri.ca/education-and-research/how-to-cite-arcgis-maps-and-data>), we have also added a reference list entry for the base layer (line 311) and a statement acknowledging the use of ArcGIS software to create the map (lines 219-220). |
| 2. Please update your Competing Interests statement. If you have no competing interests to declare, please state: “The authors have declared that no competing interests exist. | We have updated the Competing Interests statement within the submission system. |
| *Reviewer #1* | |
| Line 46: a more current reference is the FAO et al., 2020 The state of food security and nutrition in the World 2021. | Thank you to the reviewer for pointing this out. We were using the 1996 reference for the definition of food insecurity (the lack of access to sufficient safe, and nutritious food), rather than the latter part of the sentence. We have now moved the 1996 reference to the correct part of the sentence and added the 2021 reference as suggested (line 46). We have also updated the reference in line 467 to the 2021 reference. |
| Line 47: giving some examples of negative physical health outcomes will help to balance out this paragraph and can help link to the examples given in the discussion. This is referenced this in the discussion (line 409). | We have added some examples of negative physical health outcomes in lines 47-48 (reduced nutrient intake, chronic disease, and premature death). To strengthen the connection between this sentence and the discussion, we have also included some additional references related to reduced nutrient intake (line 48). |
| Line 49: In the first introductory paragraph Radimer should be included, this work was the first to highlight the relationship between FI and feelings of worry and stress and is the first FIES question (Radimer, K. L., Olson, C. M., Greene, J. C., Campbell, C. C., & Habicht, J.-P. (1992). Understanding hunger and developing indicators to assess it in women and children. Journal of Nutrition Education, 24(1), 36S-44S. https://doi.org/10.1016/S0022-3182(12)80137-3) | Thank you for this suggestion. We have added some relevant references to the sentence in line 50, including the Radimer reference. |
| Lines 50-57: states “youth are vulnerable to poor mental health” add some statistical evidence to support this as you have suggested in the discussion (line 392).This relationship would be strengthened by giving some examples: e.g. in US 2018 suicide was the second leading cause of death for youth (15-24yrs) https://www.cdc.gov/injury/wisqars/pdf/ leading_causes_of_death_by_age_group_2018-508.pdf | We have added some statistical evidence to strengthen this statement (lines 57-59). |
| Lines 50 -57: states “youth are vulnerable to FI” but this needs to be developed more, other studies find youth to be the least vulnerable to FI as they can between the homes of friends and extended families for meals, they can get part time work and have access to school food programs and food banks unlike preschool children who are confined to food sources at home.  The paragraph really focuses upon mental health, there needs to be something on the physiological impacts of FI and youth. This relationship is mentioned in the discussion (lines 365-368) and would strengthen the paper to provide some examples in the introduction. | We thank the reviewer for their suggestions.  We have strengthened the rationale as to why youth are vulnerable with respect to FI, particularly in the later years of youth when they become more independent from parental support (lines 53-56). The intent of these sentences was not to suggest that youth are one of the most vulnerable populations to FI. To reduce the strength of the statement in line 52, we have removed the word ‘particularly’. We have done the same in line 363.  Our intention was to focus on mental health in this paragraph, given that mental health is the main focus of our research question. As mentioned above, we have added some examples of negative physical health outcomes in lines 47-48 to help readers understand the physiological impacts of FI. |
| The second paragraph (Lines 50-57) explains only one direction how FI impacts mental health, it does not discuss the impact of poor mental health has upon FI. For example, when people are depressed and anxious it can impact their ability to gain and maintain employment and therefore access to food or have the energy to get and cook for themselves which can cause FI. This is mentioned in the strengths and limitations (line 427). | As the reviewer mentions, we have touched on this potential bidirectionality in the discussion section. To emphasize this point in the introduction section, we have added a sentence describing the complexity of the FI-mental health relationship (lines 50-51). |
| Line 69: The authors focus upon youth but have not identified any unique vulnerabilities of this population with regards to climate related disasters. Doing this would strengthen the relevance of the paper. | We have added a sentence that describes the unique vulnerabilities of this population (lines 76-79). We agree that this will help strengthen the relevance of the paper. |
| Line 123: The FIES was developed by the FAO, you have the reference in the discussion (45) move it up here. | Thank you for pointing this out. The reference has been added to line 135. |
| 348 -349: the case has not been made that youth are particularly vulnerable to FI and climate related disasters. When the introduction is vised add how they are vulnerable. | Please see our response to the above comment regarding line 69. |
| Line 364: please explain what “toxic stress” is and why it is relevant to the condition of FI and to youth. | Toxic stress occurs under chronic pressures (such as prolonged experiences of food insecurity) and lack of adequate supports. We have re-worded this explanation in lines 376-378. We have also added some references specific to the effects of toxic stress on the mental health of children and youth (line 379). |
| Line 367-368: There are three forms of malnutrition including under nutrition and over nutrition not all have shown a causal relationship to mental health and gut microbiome. The references used only consider mood, I would suggest either modify to “can contribute” or return to the literature. | Changed ‘contribute’ to ‘can contribute’ (line 382). |
| Line 377: Give some examples of large scale stressors and places. It is also important to recognize that many lower-income countries operate from a communal paradigm not an individual one as found in many higher income countries and perhaps that is also a contributing factor to better mental health as youth a sense of belonging and purpose. | Added examples of large-scale stressors to line 395 (poverty, conflict, and insecurity). We have also added a sentence to recognize the potential role of collectivist cultures in youth mental health (lines 391-393). |
| Line 392: in regards to the ‘dose-response’ there is an absence of physiological impacts hunger and malnutrition have upon the development of youth and rates of suicide and depression and these need to be recognized. | We have added a sentence that expands on the potential physiological mechanisms through which hunger may contribute to depression and suicide-related outcomes (lines 410-412). |
| Line 408: unless the concept of food security is included remove “availability” as shame and worried related to FI are in response to the pillar of access – money. Availability is the production side of food security not how people gain access to the food available. | Thank you for pointing this out. We have removed the word ‘availability’ from the sentence (line 428). We have chosen not to define the four pillars of food insecurity in the introduction, given that not all of these concepts are linked to the rationale and interpretation behind our work. Instead, we chose to provide the definition of food insecurity (line 45). |
| Line 418: add “youth” to the sentence. | Added (line 438). |
| *Reviewer #2* | |
| 1. The rationale for focusing on young adults? why not adults? | As per the suggestions of Reviewer #1 above (points 4, 5, and 7), we have added some additional details to our rationale for focusing on the youth population (lines 52-61, lines 74-79). In addition, we make the point in lines 61-63 that it is a relatively understudied topic. |
| 2. How the consent have been drawn from the young adults? | Added a description to lines 115-117. |
| 3. How the author drawn sample size calculation? What is the rationale behind selecting 28292 sampling data? | We thank the reviewers for their questions. As stated in lines 100-103, our sample came from the 2017 cycle of the Gallup World Poll survey, a nationally representative survey of individuals aged 15 and older from 148 countries. Gallup surveys approximately 1000 individuals from each country. From this survey, we sampled any individual between the ages of 15-24 (stated in lines 112-113). The supplementary material contains further details on how we selected the final sample of 28,929 youth from 142 countries (i.e., lists exclusions due to missing data). |
| 4. The author has not described the validity and accuracy of the study tool used to assess for FI and mental health status | In terms of the food insecurity measure, we have added some additional references that further describe the scale’s reliability and validity (lines 136-137). We have also added a sentence describing evidence for validity through strong correlations with related measures (lines 138-140).  In terms of the mental health measure, this scale was developed by Gallup itself, therefore we do not have access to detailed information on its development and validation. In lines 149-150, we report the country-level Cronbach’s alpha value that was provided by Gallup. We have now added an interpretation of this value (i.e., that this represents a satisfactory level of internal consistency). |
| 5. The variable of interest is drawn from the group level, how does the author justify the effect size? | We are in agreement with the reviewers that this is a very important question. In lines 452-458, we do mention ecological fallacy as a potential limitation of our study. The climate-related disaster variable was only available at the country level, which may not have reflected the true experiences of individuals in our sample. To avoid ecological fallacy, we interpreted the effects of this variable exclusively at the country level. |
