# Supplementary material for: Investigating the role of climate-related disasters in the relationship between food insecurity and mental health for youth aged 15–24 in 142 countries
Source: PLOS Glob Public Health. 2022 Sep 7;2(9):e0000560. doi: 10.1371/journal.pgph.0000560 (PMC10021754; doi:10.1371/journal.pgph.0000560)
Supplement: Attachment — Submitted filename: Response to Reviewers.docx [file pgph.0000560.s003.docx]

| **Reviewer Comment** | **Response** |
| --- | --- |
| *Journal Requirements* | |
| 1. Please review your reference list to ensure that it is complete and correct. If you have cited papers that have been retracted, please include the rationale for doing so in the manuscript text, or remove these references and replace them with relevant current references. Any changes to the reference list should be mentioned in the rebuttal letter that accompanies your revised manuscript. If you need to cite a retracted article, indicate the article’s retracted status in the References list and also include a citation and full reference for the retraction notice. | We have reviewed the reference list to ensure that it is complete and correct. We did not identify any retracted articles. |
| *Reviewer #1* | |
| In the introduction the addition of line 67-68 is important but does require further elaboration in another sentence or two to explain the bidirectional relationship. | We thank the reviewer for their suggestion. We have included an additional sentence to further describe the bidirectional relationship between food insecurity and mental health (lines 67-69). |
| *Reviewer #2* | |
| 1. The sample size calculation for drawing the total samples is required | Thank you. Our study was a secondary data analysis where we used all available data on the variables of interest. As such, we sampled any individual between the ages of 15-24 from the Gallup World Poll survey. The supplementary material contains further details on how we selected the final sample of 28,929 youth from 142 countries (i.e., lists exclusions due to missing data). In lines 245-246, we state that the regression analyses were ≥90% powered to detect a relative risk of 1.5 for outcomes ranging in prevalence from 20%-60%. |
| 2. The tool used for the exposure and outcome assessment has not been clearly demonstrated in terms of data collection. | We thank the reviewer for their comment. The data collection methods for the Gallup World Poll are described in lines 121-131. The data collection technique (telephone vs. face-to-face) was specific to each country, and remained consistent for all participants in that country. The supplemental material contains a list of all countries included in our analysis (starting on page 4). We have added the data collection method used by each country to this table. |
| 3. The rational for doing binomial negative regression model | Our mental health outcome was represented by discrete counts, therefore we chose to use a negative binomial regression model. We have also added a sentence to explain why we chose the negative binomial model over the Poisson model (lines 232-233). |
| 4. List of confounders are not accurate | As mentioned in lines 203-204, we chose our potential confounders based on rationale from existing literature, as well as their availability in the Gallup World Poll dataset. We have added references to the above statement to provide clarity to readers regarding the specific references that we used to identify potential confounders (line 204). In terms of confounders that we did not include, this was limited by the availability of variables in the Gallup World Poll dataset. We identify this as a limitation in the discussion section (lines 468-471).  At the end of this document, we have included a table with detailed justification for each potential confounding variable. We are open to including this table in the manuscript supplement if the reviewer believes that this would be helpful for readers. |
| 5. Discussion has to be updated after highlighting the key study outcomes. The current part of discussion seems messy. I would request author to re work on the discussion. | As stated in line 390, our analysis revealed two main findings. The first key outcome was the significant association between food insecurity and poor mental health among youth globally, and this is presented in the second paragraph of the discussion. We go on to discuss the nature and potential causes of this finding in the third and fourth paragraphs. In the fifth paragraph, we present the second key outcome: the weak yet significant interaction between food insecurity and disaster severity. The sixth and seventh paragraphs outline the main strengths and limitations of our work.  We have also addressed the reviewer’s comments on the discussion section in the PDF document. We welcome specific suggestions from the reviewer on how to re-work the discussion. |
| Additional PDF comments | Please see the attached copy for responses to all additional comments from the PDF.  Please note that any line numbers in these comments refer to those in the revised version of the manuscript, rather than the present version. |

Justification of potential confounding variables

| **Variable** | **Justification** |
| --- | --- |
| Age | The literature suggests that younger individuals experience greater FI [1,2]. Additionally, throughout the course of young adulthood individuals experience substantial physical, emotional, and social milestones, meaning that age is closely related to one’s mental health [3,4]. Age is not on the causal pathway between FI and mental health. Furthermore, all previous works that used the GWP dataset to study this relationship controlled for age in their analyses [5–11]. |
| Gender | The literature suggests that adolescent females are more likely to experience FI than males [12]. Females are also more likely to experience mental health problems [3,13]. Additionally, gender is not on the causal pathway between FI and mental health. All previous studies using the GWP dataset controlled for gender in their analyses [5–11]. |
| Urbanicity | Rural-urban differences exist in terms of FI, where prevalence is typically greater among urban young adults [14,15]. The literature also shows rural-urban differences in mental health. While some studies report a higher prevalence of mental health problems among rural dwellers compared to urban dwellers [13,16,17], others report the opposite [17,18]. Additionally, urbanicity does not lie on the FI–mental health causal pathway. Several previous works controlled for urbanicity in their modelling [5,6,8–11]. |
| No. of children in household | Having a greater number of children is associated with increased FI [1,19]. Also, young parents, in particular young mothers, are more likely to experience poor mental health [20,21]. Number of children does not lie on the causal pathway between FI and mental health. Additionally, several previous works have controlled for this variable [5,6,8]. |
| Marital status | Single adult households are more likely to experience FI [1,15]. Among young adults, marriage has shown to improve mental health in some cases [22,23]. Marital status does not fall on the causal pathway between FI and mental health. In their analyses of GWP data, Frongillo and colleagues [6] and Asfahani and colleagues [11] controlled for marital status. |
| Socioeconomic status (income, employment, education) | The literature presents a strong association between FI and poor SES among all age groups, including young adults [2,12,24]. Additionally, low SES has been frequently linked to poor mental health [13,25,26]. Given that FI itself does not lead to poor SES, this variable does not lie on the causal pathway. Further, previous works controlled for SES variables in their modelling [5,6,8–11]. The GWP contains measures of education level, employment status, and household-level income, all of which are closely related to SES. |

**Abbreviations:** food insecurity (FI), Gallup World Poll (GWP), socioeconomic status (SES).

**Note:** Confounding variables must have strong relationships with both the exposure and outcome and cannot fall on the causal pathway.

1.	Bocquier A, Vieux F, Lioret S, Dubuisson C, Caillavet F, Darmon N. Socio-economic characteristics, living conditions and diet quality are associated with food insecurity in France. Public Health Nutr. 2015 Nov;18(16):2952–61.

2.	Martinez S, Webb K, Frongillo E, Ritchie L. Food insecurity in California’s public university system: What are the risk factors? J Hunger Environ Nutr. 2017 Nov 20;13:1–18.

3.	Moreh S, O’Lawrence H. Common risk factors associated with adolescent and young adult depression. J Health Hum Serv Adm. 2016;39(2):283–310.

4.	Low NC, Dugas E, O’Loughlin E, Rodriguez D, Contreras G, Chaiton M, et al. Common stressful life events and difficulties are associated with mental health symptoms and substance use in young adolescents. BMC Psychiatry. 2012 Aug 17;12(1):116.

5.	Jones AD. Food insecurity and mental health status: A global analysis of 149 countries. Am J Prev Med. 2017 Aug 1;53(2):264–73.

6.	Frongillo EA, Nguyen HT, Smith MD, Coleman-Jensen A. Food insecurity is associated with subjective well-being among individuals from 138 countries in the 2014 Gallup World Poll. J Nutr. 2017 Mar 1;147:680–7.

7.	Frongillo EA, Nguyen HT, Smith MD, Coleman-Jensen A. Food insecurity is more strongly associated with poor subjective well-being in more-developed countries than in less-developed countries. J Nutr. 2019 Feb 1;149(2):330–5.

8.	Na M, Miller M, Ballard T, Mitchell DC, Hung YW, Melgar-Quiñonez H. Does social support modify the relationship between food insecurity and poor mental health? Evidence from thirty-nine sub-Saharan African countries. Public Health Nutr. 2019;22(5):874–81.

9.	Elgar FJ, Pickett W, Pförtner TK, Gariépy G, Gordon D, Georgiades K, et al. Relative food insecurity, mental health and wellbeing in 160 countries. Soc Sci Med. 2021 Jan 1;268:113556.

10.	Elgar FJ, Sen A, Gariépy G, Pickett W, Davison C, Georgiades K, et al. Food insecurity, state fragility and youth mental health: A global perspective. SSM - Popul Health. 2021 Jun 1;14:100764.

11.	Asfahani F, Kadiyala S, Ghattas H. Food insecurity and subjective wellbeing among Arab youth living in varying contexts of political instability. J Adolesc Health. 2019 Jan 1;64(1):70–8.

12.	Hadley C, Lindstrom D, Tessema F, Belachew T. Gender bias in the food insecurity experience of Ethiopian adolescents. Soc Sci Med. 2008 Jan 1;66(2):427–38.

13.	Alegría M, NeMoyer A, Falgàs Bagué I, Wang Y, Alvarez K. Social determinants of mental health: Where we are and where we need to go. Curr Psychiatry Rep. 2018 Sep 17;20(11):95.

14.	Belachew T, Lindstrom D, Gebremariam A, Jira C, Hattori MK, Lachat C, et al. Predictors of chronic food insecurity among adolescents in Southwest Ethiopia: A longitudinal study. BMC Public Health. 2012 Aug 3;12(1):604.

15.	Tomayko EJ, Mosso KL, Cronin KA, Carmichael L, Kim K, Parker T, et al. Household food insecurity and dietary patterns in rural and urban American Indian families with young children. BMC Public Health. 2017 Jun 30;17(1):611.

16.	Robinson LR, Holbrook JR, Bitsko RH, Hartwig SA, Kaminski JW, Ghandour RM, et al. Differences in health care, family, and community factors associated with mental, behavioral, and developmental disorders among children aged 2–8 years in rural and urban areas — United States, 2011–2012. MMWR Surveill Summ. 2017 Mar 17;66(8):1–11.

17.	Lau R, Morse CA. Health and wellbeing of older people in Anglo-Australian and Italian-Australian communities: A rural–urban comparison. Aust J Rural Health. 2008;16(1):5–11.

18.	Wang J. Rural-urban differences in the prevalence of major depression and associated impairment. Soc Psychiatry Psychiatr Epidemiol. 2004 Jan 1;39(1):19–25.

19.	Akerele D, Sanusi RA, Fadare OA, Ashaolu OF. Factors influencing nutritional adequacy among rural households in Nigeria: How does dietary diversity stand among influencers?. Ecol Food Nutr. 2017;56(2):187–203.

20.	Agnafors S, Bladh M, Svedin CG, Sydsjö G. Mental health in young mothers, single mothers and their children. BMC Psychiatry. 2019 Apr 11;19(1):112.

21.	Van Lieshout RJ, Savoy CD, Boyle MH, Georgiades K, Jack SM, Niccols A, et al. The mental health of young Canadian mothers. J Adolesc Health. 2020 Apr 1;66(4):464–9.

22.	Uecker JE. Marriage and mental health among young adults. J Health Soc Behav. 2012 Mar 1;53(1):67–83.

23.	Horwitz AV, White HR, Howell-White S. Becoming married and mental health: A longitudinal study of a cohort of young adults. J Marriage Fam. 1996;58(4):895–907.

24.	Tarasuk V, Vogt J. Household food insecurity in Ontario. Can J Public Health Rev Can Santé Publique. 2009 May 1;100:184–8.

25.	Zhang J, Mckeown RE, Hussey JR, Thompson SJ, Woods JR. Gender differences in risk factors for attempted suicide among young adults: Findings from the Third National Health and Nutrition Examination Survey. Ann Epidemiol. 2005 Feb 1;15(2):167–74.

26.	Nagasu M, Yamamoto I. Impact of socioeconomic- and lifestyle-related risk factors on poor mental health conditions: A nationwide longitudinal 5-wave panel study in Japan. PLOS ONE. 2020 Oct 9;15(10):e0240240.
